# Simultaneous and selective electrochemical determination of hydroquinone, catechol and resorcinol at poly(1,5-diaminonaphthalene)/glassy carbon-modified electrode in different media

**DOI:** 10.1039/c7ra13665j

**Published:** 2018-02-07

**Authors:** Khalid Mahmoud Hassan, Abla Ahmed Hathoot, Mohamed Fathi Abo oura, Magdi Abdel Azzem

**Affiliations:** Chemistry Research Laboratory, Physics and Mathematics Engineering Department, Faculty of Electronic Engineering, Menoufia University Egypt drkhalidhassan73@gmail.com +20 1001303945; Electrochemistry Laboratory, Chemistry Department, Faculty of Science, Menoufia University Egypt

## Abstract

The electrochemical behavior of phenolic isomers hydroquinone (HQ), catechol (CC) and resorcinol (RC) was examined in poly(1,5-diaminonaphthalene)/glassy carbon-modified electrode (P1,5-DAN/GC M.E.) by cyclic voltammetry (CV), square wave voltammetry (SWV) and chronoamperometry (CA) techniques in perchloric acid (HClO_4_) and phosphate buffer solution (PBS, pH 7.0). P1,5-DAN/GC M.E. was investigated for simultaneous determination of HQ, CC and RC in single, binary and ternary systems. Oxidation peak potentials were negatively shifted with increasing oxidation peak current for HQ, CC and RC at P1,5-DAN/GC M.E. compared with bare GC electrode. The obtained results illustrate that the former electrode exhibits better performance towards the three isomers in PBS rather than in HClO_4_ solution. The catalytic currents for different concentrations of HQ, CC and RC showed good relationship in the range of 0.1–100 μM for all analytes and low detection limits (LOD) of 0.034, 0.059 and 0.14 μM for them, respectively, in a ternary system in PBS at pH 7.0. This method has been practically applied for the detection of these isomers in tap water with acceptable results.

## Abstract 2

Electrochemical behaviors of hydroquinone, catechol and resorcinol were examined at poly(1,5-diaminonaphthalene)/glassy carbon-modified electrode by cyclic voltammetry, square wave voltammetry and chronoamperometry techniques in different media.

## Graphical abstract

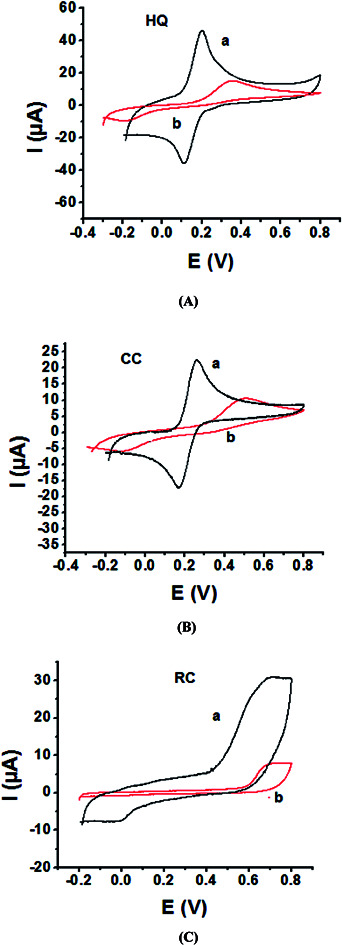

## 1. Introduction

Phenolic compounds broadly occur in the environment. Hydroquinone (HQ), catechol (CC) and resorcinol (RC) are isomers of phenolic compounds. They can be extensively used for many industrial and medicinal purposes.^1^ Phenolic compounds are slowly broken down into less harmful compounds in the natural environment causing environmental pollution. They frequently coexist together and interfere in their detection^2^ due to their similar structures and behaviors. Thus, it is essential to improve an easy and practical analytical technique for individual and simultaneous determination of these isomers. Spectrophotometry,^3,4^ gas chromatography coupled with mass spectrometry,^5^ high-performance liquid chromatography,^6,7^ chemiluminescence,^8^ fluorescence^9^ and electrochemical techniques^10–14^ had been used for the IR detection. Electrochemical procedures are desirable and interesting for the simultaneous identification due to the rapid response, low cost, high quantification and selectivity.^10^ However, the redox response of these phenolic compounds, mainly HQ and CC, often interfere, making them difficult to be determined by conventional electrodes. Recently, different modified electrodes have been suggested for simultaneous identification of HQ and CC.^2,11^ Simultaneous determination of CC, HQ and RC by electrochemical methods is not widely reported in the literature.^12–14^ It is necessary to improve modified electrodes with good catalytic response and appropriate conductivity for distinguishing these compounds simultaneously. Early, we have reported the synthesis of P1,5-DAN/GC M.E. in both aqueous and non-aqueous media.^15,16^ In addition, we had been involved in a program dealing with single and simultaneous detection of several analytes at modified electrodes prepared by electropolymerization methods.^17–19^

Up to our knowledge, the application of P1,5-DAN/GC M.E. for the simultaneous detection of HQ, CC and RC was not explored. In this work, the electrocatalytic behavior of this M.E. towards the electro-oxidation of the three analytes and its application in real water samples will be investigated. As the electrochemical redox reactions of bishydroxy benzene compounds include proton exchange, their electrochemical behavior will be examined in acidic and neutral media.

## 2. Experimental

### 2.1. Instruments and reagents

A potentiostat (PST 006) from Voltalab-Radiometer Analytical with software Model voltaMaster 4 was used for electrochemical measurements. All voltammograms were recorded with a three-electrode cell containing a 3 mm GC electrode as a working electrode, a platinum wire as an auxiliary electrode and silver/silver chloride (Ag/AgCl) as a reference one. Diamond paste 2.0 μM was used to clean the working electrode. A digital pH-meter (Woon Socket, Ri0285, USA) was used for pH measurements. HQ, CC and RC obtained from Merck and HClO_4_ 70% (AR) from Guangdong China were used as received. 1,5-DAN was purchased from Merck. Potassium dibasic phosphate (K_2_HPO_4_), potassium monobasic phosphate (KH_2_PO_4_) and hydrochloric acid (HCl) of analytical grade and bidistilled water were used.

### 2.2. Preparation of P1,5-DAN/GC M.E. and surface area measurement

P1,5-DAN was fabricated using a 1.5 mM 1,5-DAN monomer at GC electrode in 1.0 M HClO_4_ solution using cyclic voltammetry (CV) technique for 15 repeated cycles (from 0.0 to 0.8 V) at a scan rate of 0.02 V s^−1^ as illustrated in the literature.^19^

The modified electrode active surface area was calculated using the Randles–Sevcik equation.^20^ The obtained active surface areas of bare GC electrode and P1,5-DAN/GC M.E. were 0.052 cm^2^ and 0.268 cm^2^, respectively.

### 2.3. Experimental procedure

The electrochemical studies of the three phenolic compounds were performed in 5.0 mL electrochemical cell using 0.05 M HClO_4_ or 0.1 M PBS at pH 7.0 as a supporting electrolyte. Records were detected using both CV and square wave voltammetry (SWV) techniques.

### 2.4. Real samples

Real samples were supplied by acidifying tap water to 0.05 M HClO_4_ or buffering using 0.1 M PBS at pH 7.0 followed by addition of known concentrations of targets.

## 3. Results and discussion

### 3.1. Electrochemical determination of individual HQ, CC and RC at P1,5-DAN/GC M.E.

**Fig. 1:**
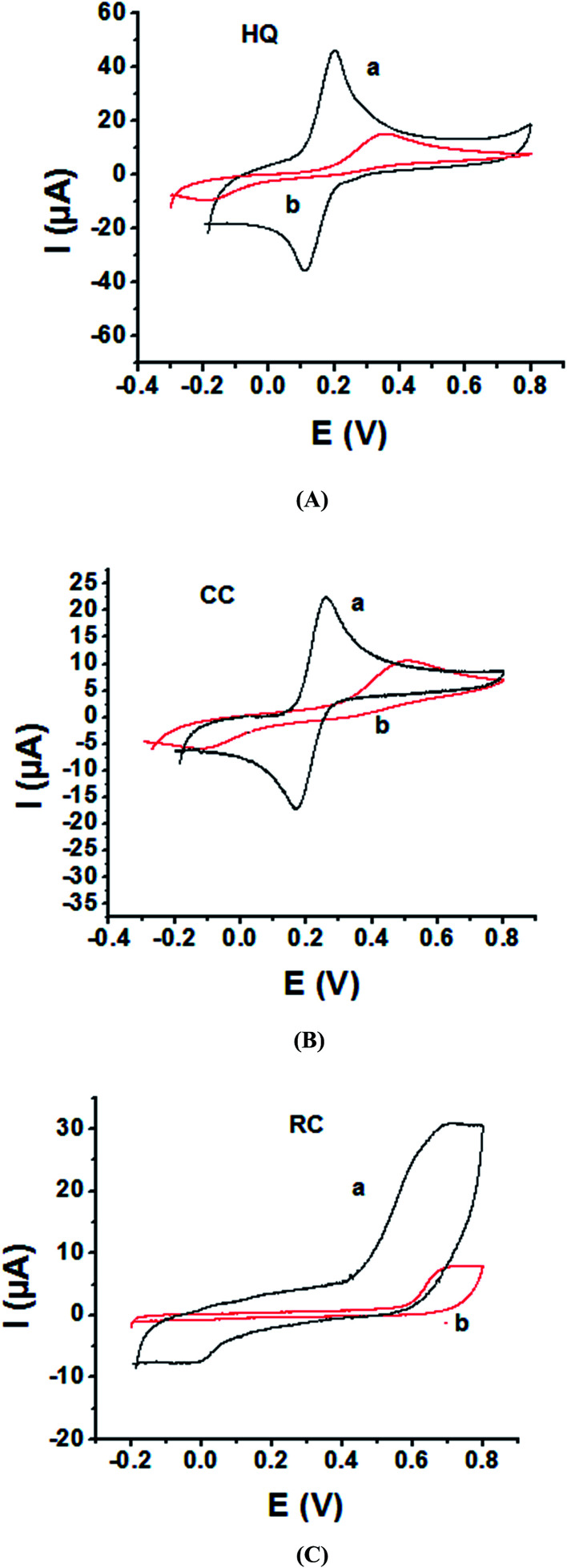
Cyclic voltammograms of 1.0 mM of (A) HQ, (B) CC and (C) RC at (a) P1,5-DAN/GC M.E. and (b) bare GC electrode in 0.05 M HClO_4_. Scan rate: 0.05 V s^−1^ in potential range from −0.03 for HQ, CC and −0.02 for RC to 0.08 V.

**Fig. 2:**
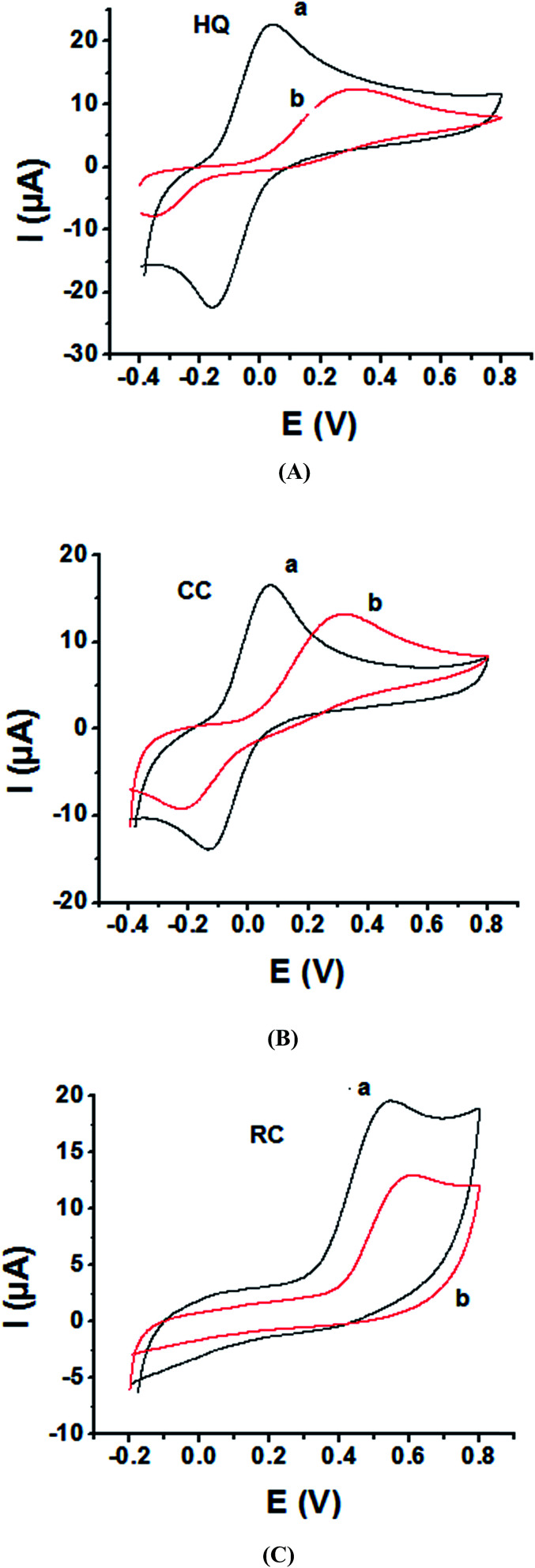
Cyclic voltammograms of 1.0 mM of (A) HQ, (B) CC and (C) RC at (a) P1,5-DAN/GC M.E. and (b) bare GC electrode in PBS (pH 7.0). Scan rate 0.05 V s^−1^ in potential range from −0.04 V for HQ, CC and −0.02 V for RC to 0.08 V.

As the electrochemical redox reactions of bishydroxybenzene compounds include proton exchange, CV studies of HQ, CC and RC at both bare GC and P1,5-DAN/GC electrodes had been performed in acidic and neutral buffer solutions. A P1,5-DAN/GC-modified electrode was prepared as mentioned in the Experimental section. Therefore, different parameters such as potential range, number of cycles, solvent selection and scan rate were studied in detail to obtain the optimum conditions for preparing the modified electrode. Dissolving 1.5 mM monomer in 1.0 M HClO_4_ solution at GC electrode by using cyclic voltammetry (CV) technique for 15 repeated cycles (from 0.0 to 0.8 V) at a scan rate of 0.02 V s^−1^ were found to be the optimum conditions. Fig. 1 and 2(A, B and C) show cyclic voltammograms recorded at a scan rate of 0.05 V s^−1^ for 1.0 mM for both HQ, CC and RC at (a) bare GC electrode and (b) P1,5-DAN/GC M.E. in 0.05 M HClO_4_ and PBS of pH 7.0, respectively. The redox peaks (*E*_pa_ and *E*_pc_), the formal potentials and the peak-to-peak separation (Δ*E*_p_) were calculated for the three isomers in both media as shown in Table 1. Obtained results indicated that the oxidations of the three targets are irreversible and undergo slow electron transfer at bare GC electrode in both acidic and neutral media. At P1,5-DAN/GC M.E., individual HQ or CC demonstrate redox systems with Δ*E*_p_ values of 0.087 and 0.093 V in HClO_4_, and 0.17 and 0.185 V in PBS pH 7.0, respectively, as presented in Fig. 1 and 2(A and B), respectively. Δ*E*_p_ for HQ and CC at P1,5-DAN/GC M.E. are much lower than that at bare GC electrode (Table 1). A significant increase in the peak current and the development of reversibility could be correlated with high surface area (0.268 cm^2^), good conductivity and electroactivity of P1,5-DAN/GC M.E. This indicates that the M.E. has excellent ability for HQ and CC determination independently without any separation or pretreatment. In case of RC (Fig. 1C and 2C), there is only one oxidation peak at 0.69 V (in HClO_4_) and 0.54 V (in PBS pH 7.0) at GC electrode. The absence of cathodic peak could be due to the lack of stability of its oxidizing state. At P1,5-DAN/GC M.E., the oxidation peak potential of RC negatively shifted to 0.60 V in HClO_4_ and 0.52 V in PBS at pH 7.0, demonstrating its effective electrocatalytic activity.

**Table 1:** Electrochemical parameters for single HQ, CC and RC determination at both GC and P1,5-DAN/GC electrodes in 0.05 M HClO_4_ and PBS pH 7.0

		Bare GC electrode						P1,5-DAN/GC M.E.					
Medium		0.05 M HClO_4_			PBS pH 7.0			0.05 M HClO_4_			PBS pH 7.0		
Analyte		HQ	CC	RC	HQ	CC	RC	HQ	CC	RC	HQ	CC	RC
Variable	*E*_Pa_/*E*_Pc_, (V)	0.353/−0.183	0.498/−0.100	0.690/—	0.296/−0.357	0.306/−0.209	0.540/—	0.198/0.111	0.260/0.167	0.603/—	0.017/−0.153	0.070/−0.115	0.525/—
	Δ*E* (V)	0.536	0.598	—	0.653	0.515	—	0.087	0.093	—	0.170	0.185	—
	Formal *E* (V)	0.268	0.299	0.335	0.326	0.257	0.335	0.154	0.213	0.301	0.085	0.094	0.262

**Fig. 3:**
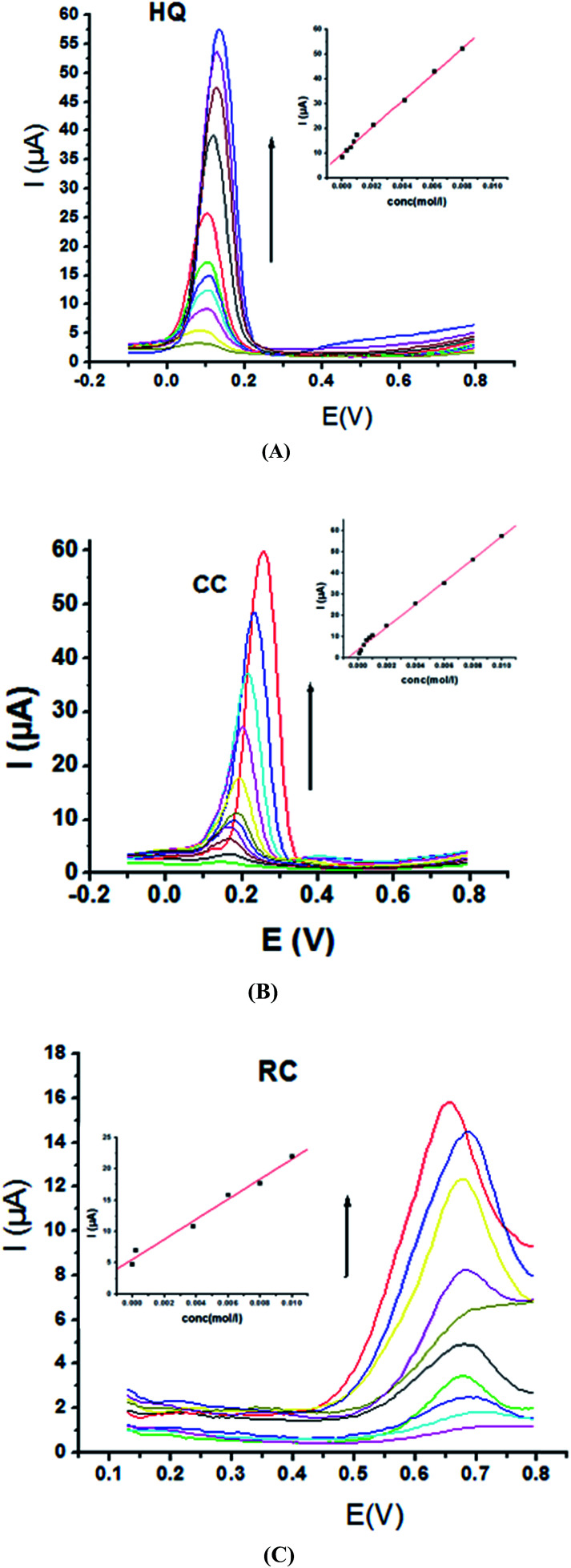
SW voltammograms of different concentrations from 0.1 mM to 10.0 mM of (A) HQ, (B) CC and (C) RC at P1,5-DAN/GC M.E. in 0.05 M HClO_4_ at a scan rate of 0.005 V s^−1^, in potential ranges – 0.01 to 0.08 V for HQ and CC while 0.01 to 0.08 V for RC, duration of 1 s, amplitude of 0.005 V and pulse of 0.025 V.

**Fig. 4:**
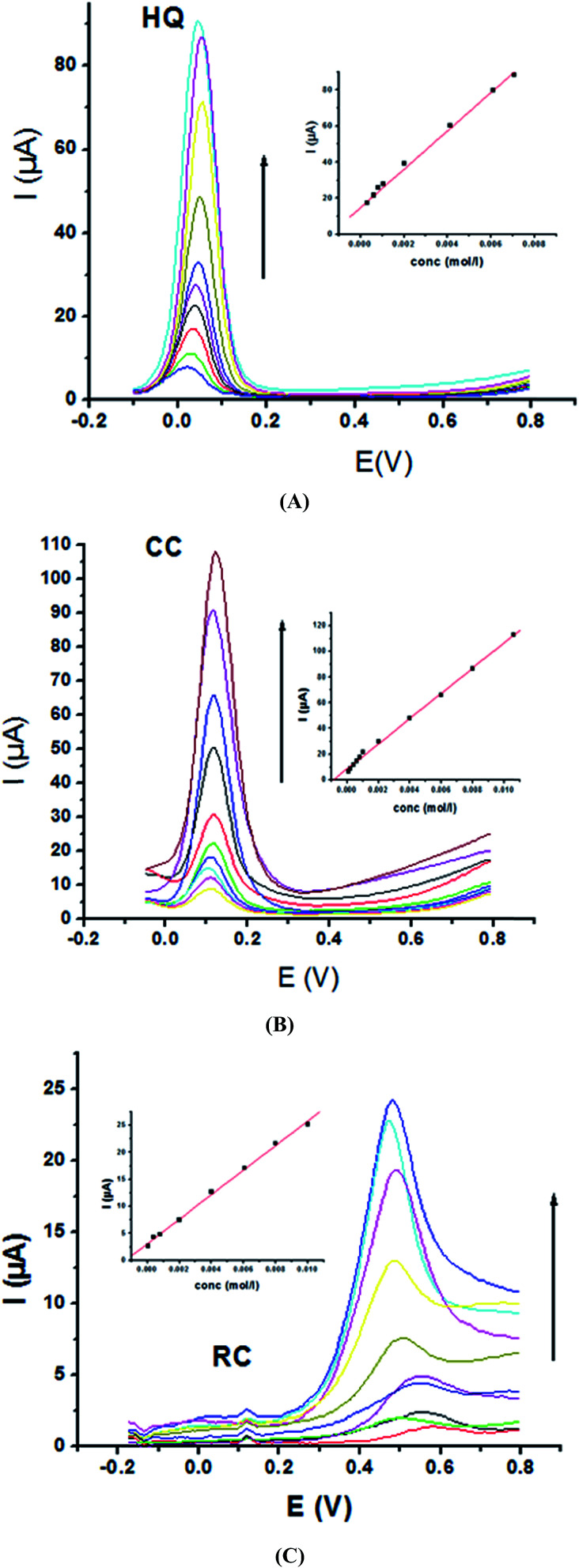
SW voltammograms of various concentrations from 0.1 mM to 10.0 mM of (A) HQ, (B) CC and (C) RC at P1,5-DAN/GC M.E. in PBS pH 7.0 at a scan rate of 0.005 V s^−1^ in potential ranges −0.01 to 0.08 V for HQ and CC while −0.02 to 0.08 V for RC, duration of 1 s, amplitude of 0.005 V and pulse of 0.025 V.

Electrochemical determination of single HQ, CC and RC at P1,5-DAN/GC M.E. was also investigated by SWV in acidic and neutral media by changing their concentrations. Fig. 3 and 4(A–C) show SW voltammograms recorded at different concentrations for the three compounds from 0.1 mM to 10.0 mM in 0.05 M HClO_4_ and PBS pH 7.0, respectively, at a scan rate of 0.005 V s^−1^. It is clear that the three phenolic isomers are oxidized at anodic potentials of 0.12, 0.22 and 0.64 V in acidic medium and 0.038, 0.10 and 0.47 V in PBS, pH 7.0, respectively. The anodic peak currents increased with the increasing analyte concentration in the range of 0.1–10 mM with calibration curves plotted as shown in (Fig. 3 and 4(A–C) insets). It was observed that the peak currents increased linearly with increasing HQ, CC, and RC concentrations. Low detection limits (LOD), low quantification limits (LOQ), linear detection ranges (LDR) and correlation coefficients (*R*) are calculated and listed in Table 2. The results indicated that the detection sensitivities for quantification of single HQ, CC and RC in PBS at pH 7.0 are better than that in acidic medium.

**Table 2:** Calibration parameters of single HQ, CC and RC at P1,5-DAN/GC M.E. in 0.05 M HClO_4_ and PBS pH 7.0

Analyte	HQ		CC		RC	
Medium	0.05 M HClO_4_	PBS pH 7.0	0.05 M HClO_4_	PBS pH 7.0	0.05 M HClO_4_	PBS pH 7.0
*E*_Pa_ (V)	0.126	0.038	0.222	0.103	0.646	0.470
LOD (μM)	0.295	0.129	0.448	0.268	0.304	0.163
LOQ (μM)	0.982	0.431	1.494	0.895	1.015	0.546
LDR (μM)	100–10 000	100–10 000	100–10 000	100–10 000	100–10 000	100–10 000
*R*	0.96	0.96	0.99	0.98	0.95	0.98

### 3.2. Simultaneous determination of HQ and CC in a binary system at P1,5-DAN/GC M.E.

**Fig. 5:**
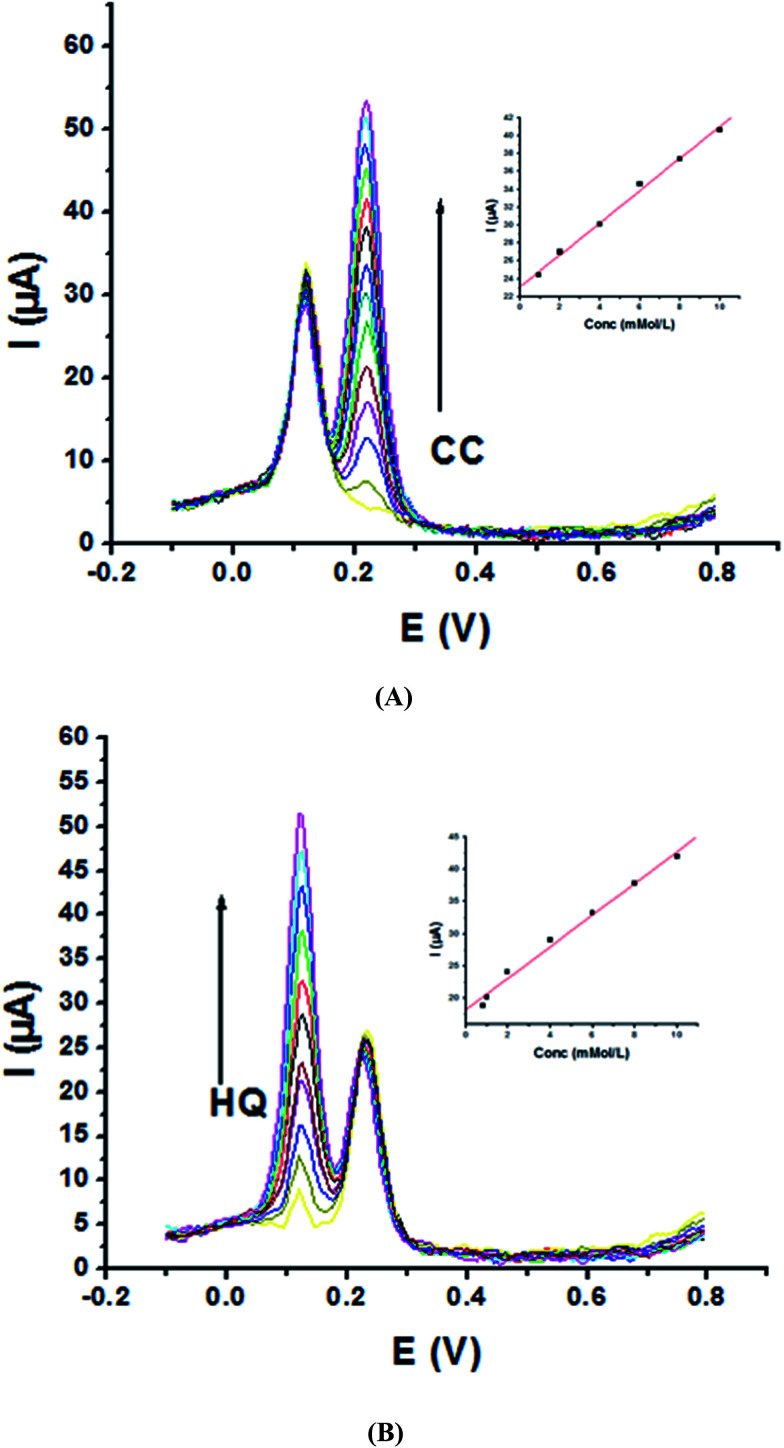
SW voltammograms of (A) 1.0 mM HQ and different concentrations of CC in the range from 0.2 to 60 mM and (B) 1.0 mM CC and different concentrations of HQ in the range from 0.2 to 20 mM at P1,5-DAN/GC M.E. in 0.05 M HClO_4_ at a scan rate of 0.005 V s^−1^ in the potential range from – 0.01 to 0.08 V, duration of 1 s, amplitude of 0.005 V and pulse of 0.025 V.

As the peak potential of HQ is close to that of CC, sensitivity and selectivity of P1,5-DAN/GC M.E. for their determination simultaneously together with the interference among each isomers on the redox behavior were examined in a binary system. Therefore, SWV were recorded in 0.05 M HClO_4_ and PBS at pH 7.0, where the concentration of one analyte increased and the other was fixed and *vice versa* at a scan rate of 0.005 V s^−1^. The obtained SW voltammograms for different concentrations of CC (0.2–60 mM) at a fixed concentration of HQ (1.0 mM) are presented in Fig. 5A. A clear peak current increased with the increasing CC concentrations (*R* = 0.99) (Fig. 5A, inset), while the oxidation peak current of HQ is approximately fixed. This behavior demonstrates that the oxidation of HQ and CC at P1,5-DAN/GC M.E. took place independently. Fig. 5B represents SW voltammograms registered at different HQ concentrations (0.2–20 mM), while the concentration of CC (1.0 mM) remained constant. Again, data demonstrated an increase in HQ oxidation peak current with its concentration (Fig. 5B, inset) with a *R* value of 0.99, while that for CC was approximately fixed. Calibration parameters of CC and HQ in binary mixtures are collected in Table 3.

**Table 3:** Calibration parameters of HQ and CC binary mixtures at P1,5-DAN/GC M.E.

Analyte	HQ		CC	
Medium	0.05 M HClO_4_	PBS pH 7.0	0.05 M HClO_4_	PBS pH 7.0
*E*_Pa_ (V)	0.122	0.010	0.222	0.110
LOD (μM)	0.761	0.146	0.247	0.094
LOQ (μM)	2.537	0.488	0.826	0.312
LDR (μM)	200–20 000	50–600	200–60 000	50–500
*R*	0.99	0.99	0.99	0.99

**Fig. 6:**
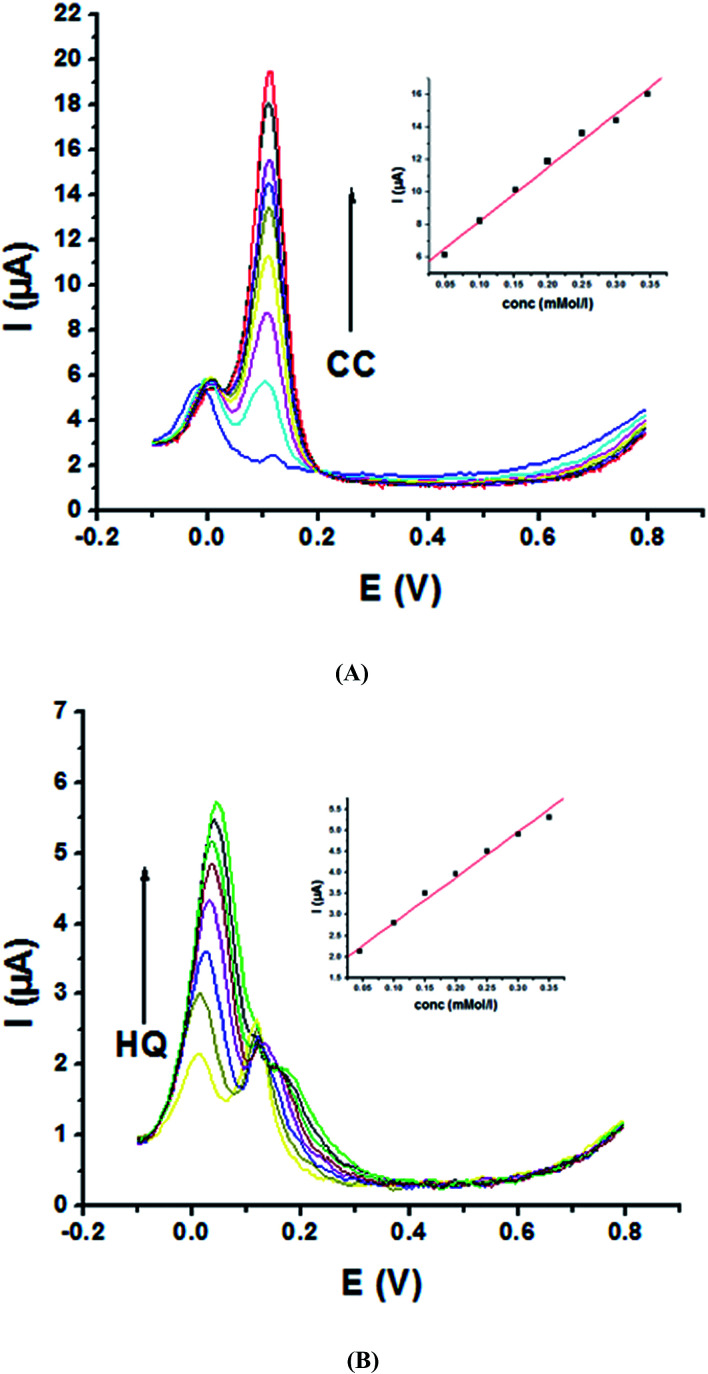
SW voltammograms of (A) 0.05 mM HQ and different concentrations of CC in the range from 0.05 to 0.5 mM and (B) 0.05 mM CC and different concentrations of HQ in the range from 0.05 to 0.6 mM at P1,5-DAN/GC M.E. in PBS (pH 7.0) at a scan rate of 0.005 V s^−1^, in the potential range from – 0.01 to 0.08 V, duration of 1 s, amplitude of 0.005 V and pulse of 0.025 V.

Moreover, P1,5-DAN/GC M.E. demonstrates good catalytic activity for the simultaneous determination of HQ and CC in a binary system in PBS. Fig. 6A shows SW voltammograms for CC different concentrations (0.05–0.5 mM) and a fixed HQ concentration (0.05 mM). Anodic peak current of CC electro-oxidation is directly proportional to its concentration with a *R* value of 0.99 (Fig. 6A, inset). Fig. 6B shows SW voltammograms for different concentrations of HQ (0.05–0.6 mM) and a fixed CC concentration (0.05 mM). A progressive increase in HQ oxidation peak current with its concentration (Fig. 6B, inset) was detected with a *R* value of 0.99, while the oxidation current of CC stays fixed. The obtained results indicated that P1,5-DAN/GC M.E. could be used to distinguish between HQ and CC; nevertheless, the concentration of CC is 15 times higher than that of HQ.

### 3.3. Simultaneous determination of HQ, CC and RC in a ternary system at P1,5-DAN/GC M.E. in acidic and neutral media

**Fig. 7:**
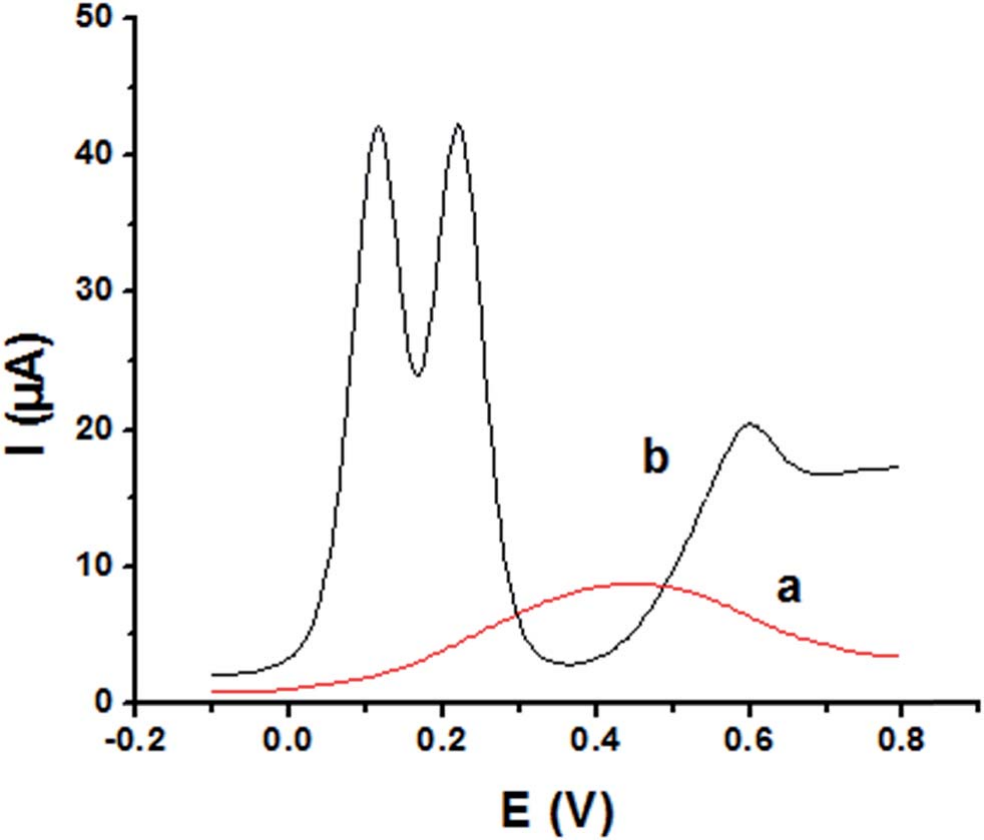
SW voltammograms of ternary system of 8.0 mM for each HQ, CC and RC in 0.05 M HClO_4_ at (a) bare GC electrode and (b) P1,5-DAN/GC M.E. at a scan rate of 0.005 V s^−1^ in the potential range from – 0.01 to 0.08 V, duration of 1 s, amplitude of 0.005 V and pulse of 0.025 V.

The main goal of this investigation is the simultaneous determination of the three isomers from their mixture solutions. Application of P1,5-DAN/GC M.E. for the simultaneous determination of HQ, CC and RC isomers in a ternary system was investigated using SWV technique in different media. Fig. 7(a and b) shows SW voltammograms for a mixture of equal concentration of 8.0 mM of each isomer in 0.05 M HClO_4_ at both bare GC and P1,5-DAN/GC electrodes at a scan rate of 0.005 V s^−1^. At bare GC electrode, the oxidation peak of the three molecules combined into one broad peak (Fig. 7a); therefore, HQ, CC and RC could not be simultaneously detected. On the other hand, at P1,5-DAN/GC M.E., three well-defined anodic peaks appeared at 0.113 V, 0.225 V and 0.599 V due to the oxidation of HQ, CC and RC, respectively (Fig. 7b). Peak potential separations for CC and HQ and RC and CC were 0.112 V and 0.374 V, respectively. P1,5-DAN/GC M.E. demonstrates its capability to discriminate between CC, HQ and RC. The increase in the separation of the peak potentials could be correlated with M.E. high surface area (0.268 cm^2^), good conductivity and electroactivity offering a sensor able to determine them simultaneously in a ternary system with high sensitivity.

**Fig. 8:**
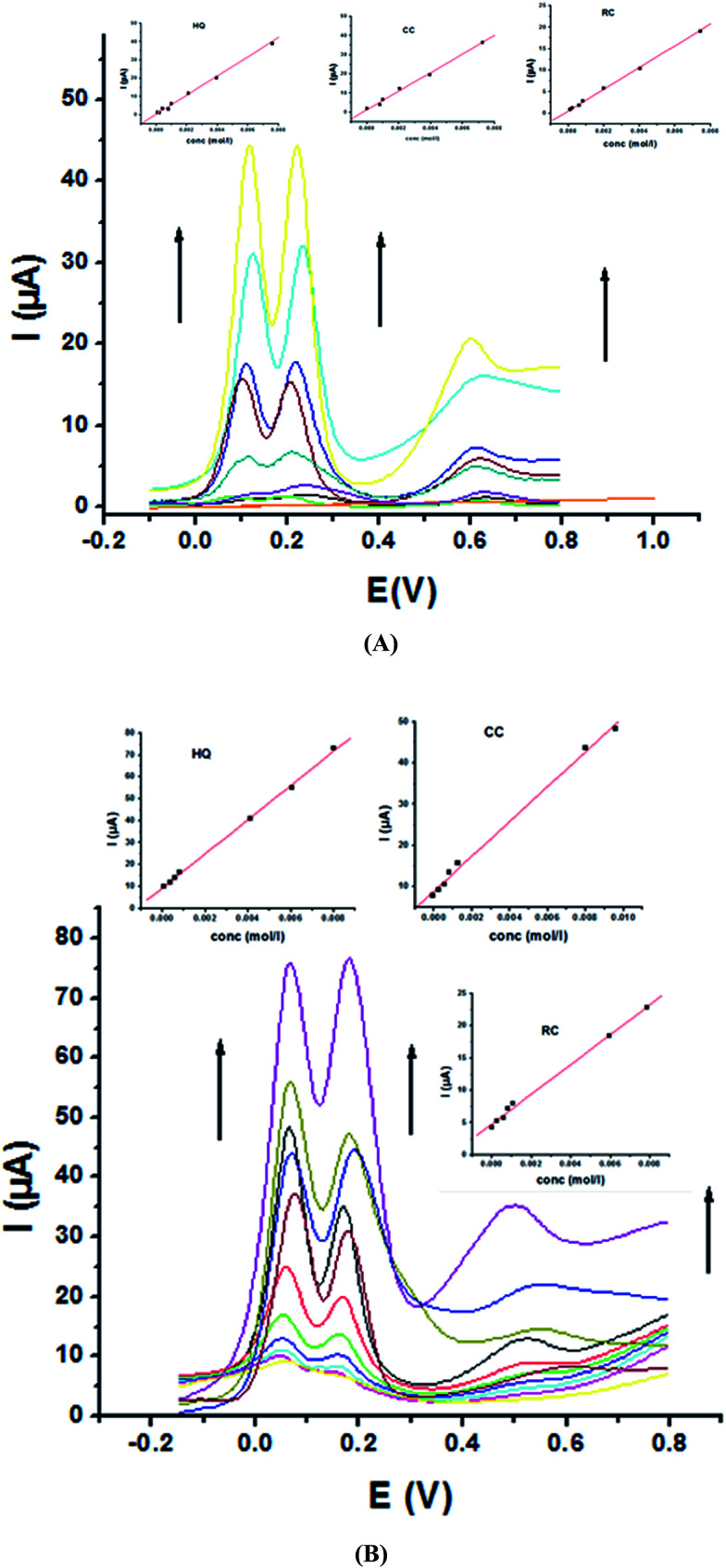
SW voltammograms of a ternary mixture of HQ, CC and RC at P1,5-DAN/GC M.E. (A) in 0.05 M HClO_4_ from 0.1 to 8.0 mM and (B) in PBS at pH 7.0 from 0.1 to 10.0 mM in a potential range from – 0.01 to 0.08 V at a scan rate of 0.005 V s^−1^, duration of 1 s, amplitude of 0.005 V and pulse of 0.025 V.

For further evaluating the feasibility of our modified electrode for CC, HQ and RC simultaneous determination, the present system was examined to determine the three isomers by simultaneously changing their concentration in acidic and neutral media (Fig. 8A and B). Increasing concentrations of ternary mixture components shows an increase in their oxidation peak currents with a linear performance in both media (Fig. 8, insets), showing the capability of P1,5-DAN/GC M.E. for their simultaneous selective and sensitive determination. This M.E. intensified the peak current and increased the efficiency of the catalytic separation of the coexistence of HQ, CC and RC. LOD, LDR and *R* were calculated in Table 4. It is important to mention that Fig. 4(C) and 8(C) demonstrate that *E*_pa_ for RC changed with its changing concentration. This observation resembles that present for DPV of 50 μM of HQ, CC and RC of different concentrations in 0.1 M PBS pH 7.0 at Au–Pd nanoflower/reduced graphene oxide nanocomposite.^21,22^

**Table 4:** Calibration parameters for simultaneous determination of HQ, CC and RC (in ternary mixtures) at P1,5-DAN/GC M.E. in 0.05 M HClO_4_ and PBS (pH 7.0) changing the three analyte concentrations

Analyte	HQ		CC		RC	
Medium	0.05 M HClO_4_	PBS pH 7.0	0.05 M HClO_4_	PBS pH 7.0	0.05 M HClO_4_	PBS pH 7.0
*E*_Pa_ (V)	0.113	0.059	0.225	0.175	0.599	0.530
LOD (μM)	0.054	0.034	0.069	0.059	0.16	0.14
LOQ (μM)	0.178	0.114	0.233	0.196	0.533	0.46
LDR (μM)	100–8000	100–10 000	100–8000	100–10 000	100–8000	100–10 000
*R*	0.99	0.99	0.99	0.99	0.99	0.99

### 3.4. Chronoamperometric study

**Fig. 9:**
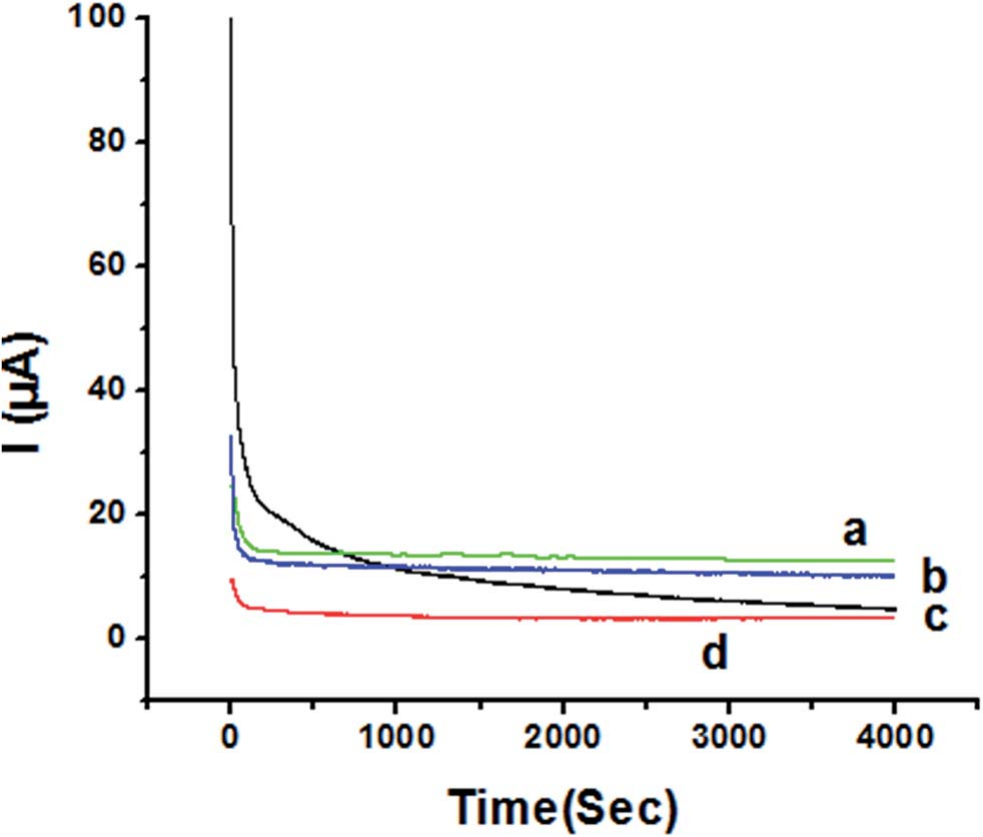
Chronoamperometry of 1.0 mM (a) HQ in PBS at pH 7.0 (b) CC in PBS at pH 7.0 (c) HQ in 0.05 M HClO_4_ (d) CC in 0.05 M HClO_4_ at P1,5-DAN/GC M.E. at applied potentials of −0.057 V, 0.011 V, 0.123 V and 0.262 V, respectively.

The activity of P1,5-DAN/GC M.E. for the determination of 1.0 mM HQ and CC in both 0.05 M HClO_4_ and PBS pH 7.0 media was investigated by chronoamperometry (CA) technique at applied potentials of −0.057 V, 0.011 V, 0.123 V and 0.262 V, respectively, for a period of 4000 seconds as shown in Fig. 9. Applying potential corresponding to each isomer, a steady decrease in currents was observed within the first few minutes for HQ and CC in PBS pH 7.0, followed by establishment of nearly constant currents at longer times. In case of acidic medium, HQ current decreased to about its half value to reach a steady state, while CC showed a constant current value. Current decreasing with time could be attributed to the intermediate poisoning species accumulated during the oxidation process.^18^ The catalytic activity was found to be greater in PBS at pH 7.0 than that in 0.05 M HClO_4_ and follows the order HQ in PBS > CC in PBS > HQ in HClO_4_ > CC in HClO_4_.

### 3.5. Mechanism of electrochemical oxidation of HQ, CC and RC

**Scheme 1:**
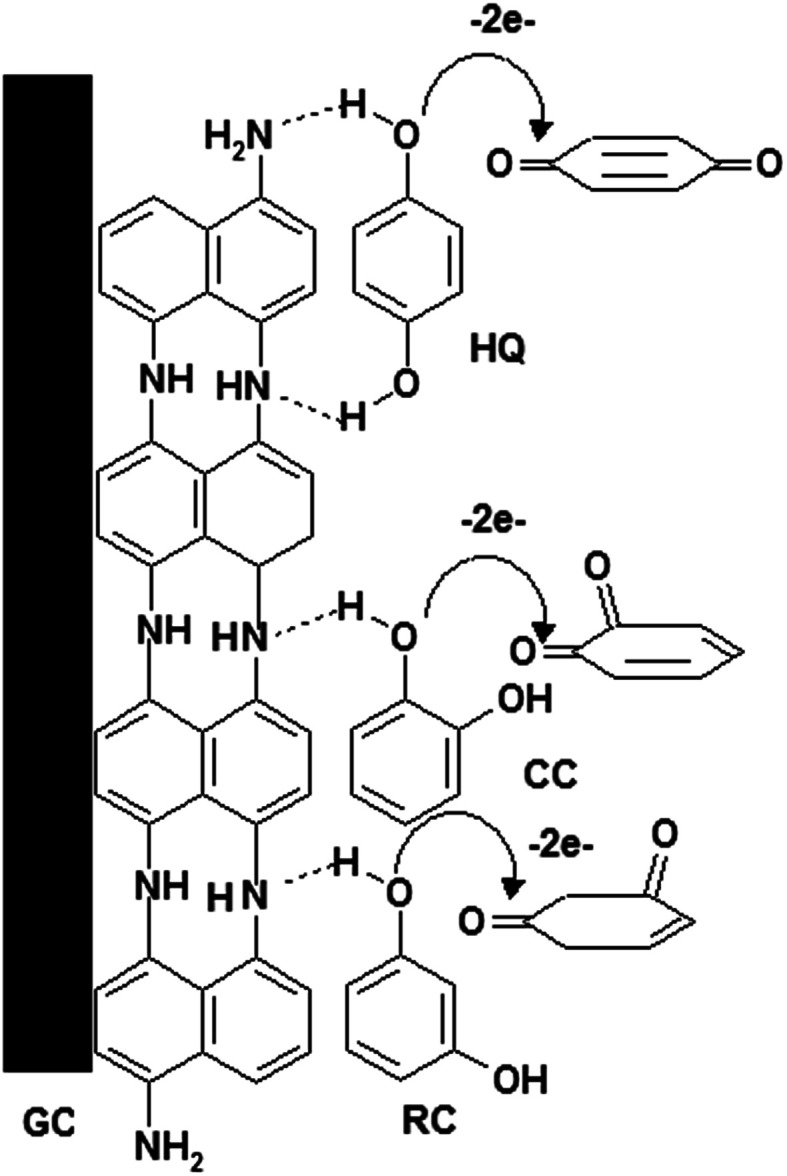
Oxidation mechanism of HQ, CC and RC at P1,5-DAN/GC M.E.

Based on foregoing results, the mechanism of the electro-oxidation of HQ, CC and RC could be suggested as shown in Scheme 1. P1,5-DAN film contains several imino groups (–NH),^23^ forming two hydrogen bonds with two hydroxyl groups of the HQ^16,20^ and one hydrogen bond with CC and RC compounds. This could lead to a decrease in the hydroxyl bond energies assisting electron transfer through O–H⋯N.^24^

Finally, Table 5 compares different response characteristics for the electrochemical detections of HQ, CC and RC at P1,5-DAN/GC M.E. and different modified electrodes reported in literatures. It was observed that the current electrode provides good catalytic behavior compared with other electrodes.

**Table 5:** Electrochemical detection of HQ, CC and RC at different modified electrodes

		Linear range (μM)			LOD (μM)			Ref.
Electrode	Method	HQ	CC	RC	HQ	CC	RC	
Graphene oxide doped poly(3,4-ethylenedioxythiophene)/GCE	DPV	2.5–200	2–400	—	1.6	1.6	—	25
Poly(diallyldimethylammonium chloride) functionalized graphene/GCE	DPV	1–500	1–400	—	0.2	0.25	—	26
Poly(3,4-ethylenedioxythiophene)/AuE	CV	0.1–49	0.091–98	—	0.0001	0.0009	—	27
MWCNTs–PDDA–GR	DPV	0.5–400	0.5–400	—	0.002	0.0018	—	28
Graphene–chitosan/GCE	DPV	1–400	1–550	1–300	0.75	0.75	0.75	29
Graphene-doped CILE	DPV	10–400	10–300	1–170	1.8	0.7	0.4	30
PANI/MnO_2_	DPV	0.2–100	0.2–100	0.2–100	0.13	0.16	0.09	31
P1,5-DAN^a^/GC	SWV	0.1–100	0.1–100	0.1–100	0.034	0.059	0.14	This work

a In PBS at pH 7.0.

### 3.6. Application in real samples

The investigated system was validated for the simultaneous determination of HQ, CC and RC in real samples such as local tap water, underground water and mineral water. HQ and CC were not detected in real samples because they are below the detection limits. Therefore, samples were spiked with equal concentrations of 50 μM of HQ, CC, and RC. By using the standard addition method, HQ, CC and RC were determined and the obtained results are tabulated in Table 6, indicating that the recovery values are in the range from 95 to 115%.

**Table 6:** Determination of HQ, CC and RC in real samples

Sample	Added μM	HQ		CC		RC	
		Found recovery μM	(%)	Found recovery μM	(%)	Found recovery μM	(%)
Underground water	50	49	98	47.5	95	50	100
Mineral water	50	54	108	50	100	57.5	115
Tap water	50	56	112	51.3	103	49	98

### 3.7. Interfering and stability studies

The influence of different substances present in environmental water samples on the simultaneous determination of phenolic compounds was examined. Therefore, different solutions containing 1.0 mM of HQ, CC and RC were analyzed in the individual presence of different concentrations for different salts. Therefore, distilled water samples containing 1.35 M HPO_4_^2−^, 0.565 M Cu^2+^, 0.90 M Na^+^, 1.35 M K^+^, 0.90 M SO_4_^2−^ and 0.90 M F^−^ ions in addition to 0.45 M glucose and 0.35 M urea were spiked with specific concentrations of HQ, CC and RC and subjected to analysis. The obtained results were compared with those obtained previously for individual HQ, CC and RC determinations, confirming the absence of cations and anions interfering. It is clear that a 900-fold concentration of Na^+^, 1350-fold of K^+^, 565-fold Cu^2+^, 900-fold SO_4_^2−^, 1000-fold F^−^, 675-fold HPO_4_^2−^, 450-fold glucose and 350-fold urea had no influence on the oxidation peaks of HQ, CC and RC (signal change ≤5%).

A long-term storage stability of P1,5-DAN/GC M.E. was tested by storing the M.E. at room temperature. After 15 days, it was observed that peak current intensities of HQ, CC and RC decreased only by 10%, 10% and 35%, respectively.

## 4. Conclusion

P1,5-DAN/GC M.E. was prepared by electropolymerization method. CV, SWV and CA techniques were applied for HQ, CC and RC analysis in single, binary and ternary systems. The electrocatalytic activity towards electro-oxidation of these analytes is enhanced at P1,5-DAN/GC M.E. and resolves the overlapping anodic peaks between HQ and CC in binary and ternary mixtures. LOD values of HQ, CC and RC in PBS at pH 7.0 were as low as 0.034, 0.059 and 0.14 μM, respectively, using SWV technique. Individual and simultaneous recovery of bisphenols are done at P1,5-DAN/GC M.E. with high sensitivity, good selectivity and low recovery limits. Moreover, the stable response and no fouling of the electrode surface are observed.

## Conflicts of interest

There are no conflicts to declare.

## Supplementary Material

## References

[cit1] Wang J., Park J.-N., Wei X.-Y., Lee C. W. (2003). Chem. Commun..

[cit2] Wang L., Huang P., Bai J., Zhao Y. (2007). Int. J. Electrochem. Sci..

[cit3] Nagaraja P., Vasantha R. A., Sunitha K. R. (2001). J. Pharm. Biomed. Anal..

[cit4] Nagaraja P., Vasantha R. A., Sunitha K. R. (2001). Talanta.

[cit5] Deceuninck Y., Bichon E., Durand S., Bemrah N., Zendong Z., Morvan M. L., Marchand P., Dervilly-Pinel G., Antignac J. P., Leblanc J. C., Le Bizec B. (2014). J. Chromatogr. A.

[cit6] Lee B. L., Ong H. Y., Shi C. Y., Ong C. N. (1993). J. Chromatogr. A.

[cit7] Cui H., He C., Zhao G. (1999). J. Chromatogr..

[cit8] Cui H., Zhang Q., Myint A., Ge X., Liu L. (2006). J. Photochem. Photobiol.

[cit9] Pistonesi M. F., Di Nezio M. S., Centurion M. E., Palomeque M. E., Lista A. G., Fernandez Band B. S. (2006). Talanta.

[cit10] Ahammad A. J. S., Nath N. C. D., Xu G.-R., Kim S., Lee J.-J. (2011). J. Photochem. Photobiol.

[cit11] Feng X., Shi Y., Hu Z. (2011). Mater. Chem. Phys..

[cit12] Yin H., Zhang Q., Zhou Y., Ma Q., Liu T., Zhu L., Ai S. (2011). Electrochim. Acta.

[cit13] Ma L., Zhao G.-C. (2012). Int. J. Electrochem..

[cit14] Anu Prathap M. U., Satpati B., Srivastava R. (2013). Sens. Actuators, B.

[cit15] Abdel Azzem M., Yousef U. S., Limosin D., Pierre G. (1994). Synth. Met..

[cit16] Abdel-Azzem M., Yousef U. S., Pierre G. (1998). Eur. Polym. J..

[cit17] Hassan K. M., Elhaddad G. M., Abdel Azzem M. (2014). J. Electroanal. Chem..

[cit18] Hassan K. M., Abdel Azzem M. (2015). J. Appl. Electrochem..

[cit19] Hassan K. M., Hathoot A. A., Ashour W. F. D., Abdel-Azzem M. (2015). J. Solid State Electrochem..

[cit20] Ahammad A. J. S., Rahman M. M., Xu G.-R., Kim S., Lee J.-J. (2011). Electrochim. Acta.

[cit21] Peifeng Bai G. F., Li F. (2011). Mater. Lett..

[cit22] Zhimin Liu Z. W., Cao Y., Jing Y., Liu Y. (2011). Sens. Actuators, B.

[cit23] Hathoot A. A., Fahmy M. E., Abdel Azzem M. (2013). Int. J. Chem. Mat. Sci..

[cit24] Hathoot A. A., Hassan K. M., Essa W. A., Abdel-Azzem M. (2017). J. Iran. Chem. Soc..

[cit25] Prabhu P., Suresh Babu R., Sriman Narayanan S. (2011). Sens. Actuators, B.

[cit26] Sharp M., Petersson M., Edstrom K. (1979). ýJ. Electroanal. Chem..

[cit27] Chandra U., Kumara Swamy B. E., Gilbert O., Sherigara B. S. (2010). Electrochim. Acta.

[cit28] Laviron E. (1979). J. Electroanal. Chem..

[cit29] Peng J., Gao Z.-N. (2006). Anal. Bioanal. Chem..

[cit30] Zhang Y., Zheng J. B. (2007). Electrochim. Acta.

[cit31] Wang Y., Rui Y., Li F., Li M. (2 014). Electrochim. Acta.

